# High‐grade myofibroblastic sarcoma of the pleura: A case report and literature review

**DOI:** 10.1111/1759-7714.13613

**Published:** 2020-08-19

**Authors:** Ruizhi Zhao, Jianyang Wang, Hongtu Zhang, Yihebali Chi, Nan Bi

**Affiliations:** ^1^ Department of Radiation Oncology, National Cancer Center/National Clinical Research Center for Cancer/Cancer Hospital Chinese Academy of Medical Sciences & Peking Union Medical College Beijing China; ^2^ Department of Pathology, National Cancer Center/National Clinical Research Center for Cancer/Cancer Hospital Chinese Academy of Medical Sciences & Peking Union Medical College Beijing China; ^3^ Department of Medical Oncology, National Cancer Center/National Clinical Research Center for Cancer/Cancer Hospital Chinese Academy of Medical Sciences & Peking Union Medical College Beijing China

**Keywords:** Chemoradiotherapy, high‐grade myofibroblastic sarcoma, pleura

## Abstract

High‐grade myofibroblastic sarcoma (HGMS) is a rare cancer that has high recurrence and metastatic rates. Here, we report the first case of HGMS originating from the pleura. Based on the findings of pleural biopsy, pathological examination and immunohistochemical staining, grade III myofibroblastic sarcoma (MS) was diagnosed. The patient underwent eight cycles of chemotherapy (epirubicin and ifosfamide), followed by radiotherapy. As of May 2020, the patient had been followed for six months and no tumor progression had occurred.

**Key points:**

This is the first report of high‐grade myofibroblastic sarcoma originating from the pleura. The patient was treated via nonsurgical strategies, including chemotherapy and radiotherapy.

## Graphical abstract

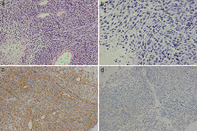

High‐grade myofibroblastic sarcoma (HGMS) is a rare disease. This is the first case diagnosed with HGMS originating from the pleura that was nonsurgically treated.

## Introduction

Malignant sarcoma consisting of myofibroblasts or exhibiting myfibroblastic differentiation is diagnosed as myofibroblastic sarcoma (MS). MS is a rare disease that typically originates in the head and neck, extremities, and the trunk.^1^ MS can be divided into low‐, intermediate‐ and high‐grade types according to the morphologic features.^2^, ^3^ The former two types together are classified as low‐grade myofibroblastic sarcoma by the WHO, but high‐grade MS (HGMS), which is also called pleomorphic myofibrosarcoma,^2^ has not as yet been included in the WHO classification. HGMS is diagnosed by cytomorphological analysis and immunophenotyping, and has higher recurrence and metastatic rates than low‐grade MS.^2^ Previous studies on HGMS are rare, and to the best of our knowledge, no case of HGMS in the pleura has been reported in the English‐language literature. Therefore, here we report the first case of HGMS originating from the pleura.

## Case report

**Figure 1:**
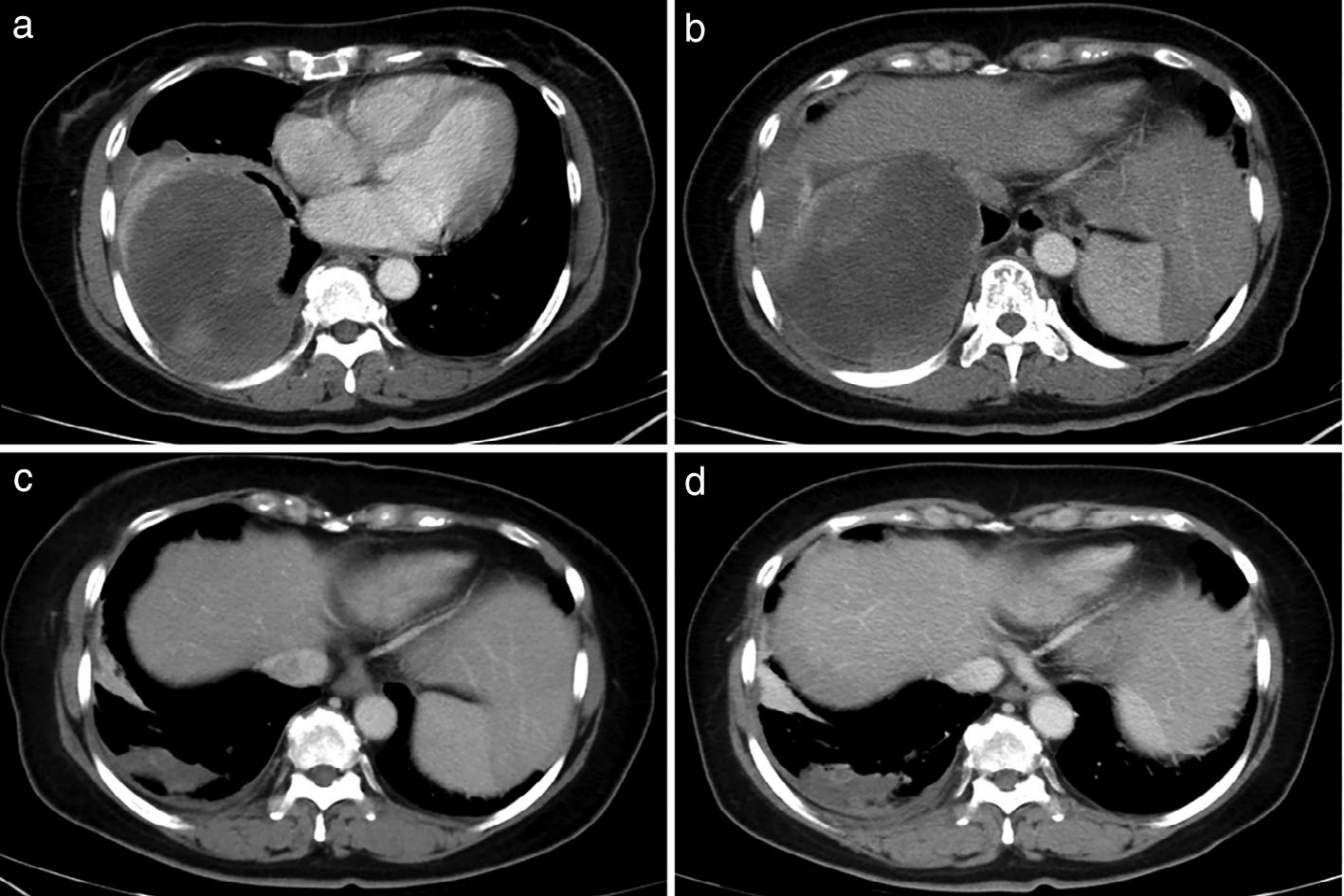
Enhanced CT scan. (**a** and **b**) A clear boundary appeared and heterogeneous enhanced soft tissue mass in the right pleura, with the maximum diameter 10.9 × 9.5 cm before chemotherapy. (**c**) The lesion shrunk to 5.2 × 2.0 cm after eight cycles chemotherapy. (**d**) The tumor was stable one month after RT.

**Figure 2:**
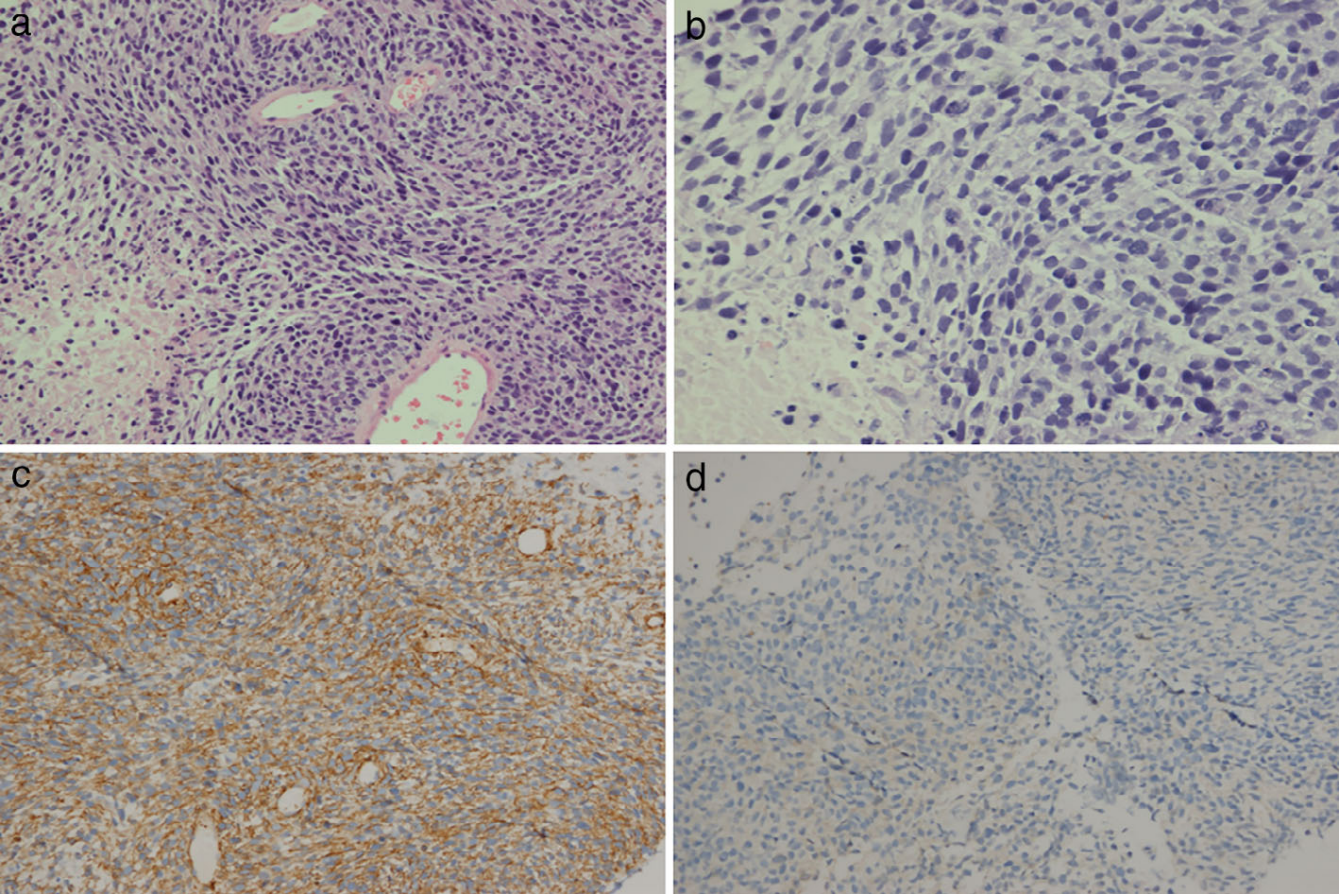
Microscopic findings. (**a**/**b**) hematoxylin‐eosin staining. (**c**) SMA was positive. (**d**) Desmin was negative.

In November 2018, a 57‐year‐old female patient presented to our hospital with a two‐month history of cough and shortness of breath. Her medical records revealed that she did not have a history of tuberculosis, malignancy, or chemoradiotherapy. Whole‐body positron emission tomography/computed tomography (PET/CT) scan revealed thickened right pleura (standard uptake value [SUV] = 23) and a hypermetabolic mass (10.9 × 9.5 cm) with a clear boundary in the right lung (max SUV = 29) (Fig 1a & b). The level of the serum tumor markers progastrin‐releasing peptide (ProGRP) and neuron‐specific enolase (NSE) was 71.66 pg/mL (upper normal limit, 69.2) and 68.99 ng/mL (upper normal limit, 16.3). The other markers were within the normal range. Pleural biopsy was performed, and pathological examination of the biopsy sample showed spindle cell sarcoma in the right pleura. Immunohistochemical staining revealed that the sample was positive for smooth muscle actin (SMA) (2+, Fig 2c), CD99 (2+), and Bcl‐2 (2+), and negative for desmin (Fig 2d), ALK, CD31, CD34, CR, MyoDI, S‐100, and WT1. Additionally, the Ki67 value was 70% (Fig 2a & b). Based on these findings, the patient was diagnosed with grade III MS.

After consultation with a multidisciplinary team, the patient was administered eight cycles of chemotherapy with epirubicin (100 mg day 1) and ifosfamide (2 g day 1) every 21 days. CT evaluation after chemotherapy showed a partial response: the lesion had shrunk to 5.2 × 2.0 cm (Fig 1c). The patient was then administered radiotherapy. The planning gross target volume (PGTV) was determined by delineating the internal gross tumor volume (IGTV) on four‐dimensional CT simulation images and expanding it by 3 mm. The clinical tumor volume (CTV) was determined by delineating the pleural region involving the tumor before chemotherapy. The planning target volume (PTV) was created by 4‐mm isotropic expansion of the IGTV and CTV. TOMO therapy was used to deliver a total dose of 50.4 Gy to the PGTV in 28 fractions, and a dose of 60.2 Gy to the PTV in 28 fractions.

One month after radiotherapy, an enhanced CT scan showed that the tumor had not progressed (Fig 1d). However, the ProGRP and NSE levels had normalized. After six‐month follow‐up, of May 2020, no tumor progression was noted.

## Discussion

This is the first report of HGMS in the pleura. HGMS is usually immunopositive for SMA, muscle‐specific actin, vimentin, and fibronectin,^1^, ^2^, ^3^, ^4^, ^5^, ^6^, ^7^, ^8^, ^9^, ^10^, ^11^ and it is occasionally positive for desmin.^2^ Further, it is immunonegative for h‐caldesmon, CK, laminin, S100, and CD34. In our case, the tumor was positive for SMA and negative for desmin, S100, CR, and CD34. Thus, the immunohistochemical findings are similar to those reported in the literature.

Surgical excision with wide margins is the most effective definitive treatment for MS, but adjuvant therapies, including chemotherapy and radiotherapy, are also considered.^1^, ^12^, ^13^ In a report by Cai and colleagues^13^ on 46 cases of MS in the head and neck region, four patients with HGMS, who had undergone partial excision, experienced relapse and died. Wen *et al*.^14^ and Anastasiou *et al*.^15^ reported two cases of HGMS in two uncommon sites—the liver and the paratesticular soft tissues. Both patients underwent surgical resection and did not require any postoperative treatment. No recurrence was recorded at the six‐month follow‐up. In 2008, Seiji *et al*.^16^ reported a case of primary HGMS in the pericardium that was treated with thoracotomy, followed by postoperative chemotherapy (one cycle of doxorubicin and ifosfamide) and radiotherapy (50 Gy) (which were necessitated by incomplete resection). The residual tumor gradually regressed, and compression of the left atrium was relieved. No progression or adverse effects were noted till six months after the surgery. The present case is the first case of HGMS arising from the pleura in which only chemotherapy and RT were administered, and surgical treatment was not performed. The tumor responded well to treatment, and no relapse occurred for up to four months after RT.

There are no standard guidelines for the treatment of HGMS, as it a rare malignant tumor. Further, there is limited clinical information on the outcomes of nonsurgical treatment, such as chemotherapy and RT. Therefore, this case provides new information that could potentially be useful for similar rare cases.

## Disclosure

None of the authors have any conflicts of interest to report.
